# Differential regulation of PGC-1α expression in rat liver and skeletal muscle in response to voluntary running

**DOI:** 10.1186/1743-7075-7-36

**Published:** 2010-04-30

**Authors:** Renata Matiello, Rosa T Fukui, Maria ER Silva, Dalva M Rocha, Bernardo L Wajchenberg, Salman Azhar, Rosa F Santos

**Affiliations:** 1 Laboratory of Medical Investigation, LIM-18, Division of Endocrinology and Metabolism, Hospital of Clinics, School of Medicine, University of Sao Paulo. Av Dr Arnaldo, 455, 3rd floor, room 3324, Sao Paulo, 01246-903, Brazil; 2 Geriatric Research, Educational and Clinical Center, VA Palo Alto Health Care System, Palo Alto, California, 94304, USA and Stanford University, Stanford, California, 94305, USA

## Abstract

**Background:**

The beneficial actions of exercise training on lipid, glucose and energy metabolism and insulin sensitivity appear to be in part mediated by PGC-1α. Previous studies have shown that spontaneously exercised rats show at rest enhanced responsiveness to exogenous insulin, lower plasma insulin levels and increased skeletal muscle insulin sensitivity. This study was initiated to examine the functional interaction between exercise-induced modulation of skeletal muscle and liver PGC-1α protein expression, whole body insulin sensitivity, and circulating FFA levels as a measure of whole body fatty acid (lipid) metabolism.

**Methods:**

Two groups of male Wistar rats (2 Mo of age, 188.82 ± 2.77 g BW) were used in this study. One group consisted of control rats placed in standard laboratory cages. Exercising rats were housed individually in cages equipped with running wheels and allowed to run at their own pace for 5 weeks. At the end of exercise training, insulin sensitivity was evaluated by comparing steady-state plasma glucose (SSPG) concentrations at constant plasma insulin levels attained during the continuous infusion of glucose and insulin to each experimental group. Subsequently, soleus and plantaris muscle and liver samples were collected and quantified for PGC-1α protein expression by Western blotting. Collected blood samples were analyzed for glucose, insulin and FFA concentrations.

**Results:**

Rats housed in the exercise wheel cages demonstrated almost linear increases in running activity with advancing time reaching to maximum value around 4 weeks. On an average, the rats ran a mean (Mean ± SE) of 4.102 ± 0.747 km/day and consumed significantly more food as compared to sedentary controls (*P *< 0.001) in order to meet their increased caloric requirement. Mean plasma insulin (*P *< 0.001) and FFA (*P *< 0.006) concentrations were lower in the exercise-trained rats as compared to sedentary controls. Mean steady state plasma insulin (SSPI) and glucose (SSPG) concentrations were not significantly different in sedentary control rats as compared to exercise-trained animals. Plantaris PGC-1α protein expression increased significantly from a 1.11 ± 0.12 in the sedentary rats to 1.74 ± 0.09 in exercising rats (*P *< 0.001). However, exercise had no effect on PGC-1α protein content in either soleus muscle or liver tissue. These results indicate that exercise training selectively up regulates the PGC-1α protein expression in high-oxidative fast skeletal muscle type such as plantaris muscle.

**Conclusion:**

These data suggest that PGC-1α most likely plays a restricted role in exercise-mediated improvements in insulin resistance (sensitivity) and lowering of circulating FFA levels.

## Background

Insulin resistance, which reflects an impaired response of glucose transport to insulin, is an early characteristic in the development of type 2 diabetes [1,2]. Together with hyperinsulinemia, insulin resistance contributes to development of a cluster of interdependent metabolic abnormalities [3-6] that in aggregate increase the risk of cardiovascular disease by about 2-fold and raise the risk for type 2 diabetes by approximately 5-fold [5]. These metabolic abnormalities have been referred to by a variety of names in the past [4], but are now commonly called 'metabolic syndrome' [4-6]. The dramatic increase in the prevalence of obesity that reflects overnutrition, global adaptation of the Western type of diet along with increased consumption of refined sugars (fructose), and sedentary lifestyles, has led to a marked increase in metabolic syndrome and type 2 diabetes [4,7-12]. Insulin resistance is now considered a direct consequence of obesity-associated exposure of tissues to elevated dietary nutrients, resulting in accumulation of toxic metabolic by-products including lipid metabolites in insulin-sensitive tissues, such as the liver, skeletal muscle, and fat that become insulin resistant [13-17]. Extensive evidence now also points to a strong association between hepatic and skeletal muscle insulin resistance and dysregulation of whole body glucose homeostasis and fasting hyperglycemia in type-2 diabetes [13-15,17]. The insulin sensitizing drugs such as thiazolidinediones and metformin that specifically target insulin resistance are currently in use to treat type-2 diabetes, but other treatment regimens, namely dietary modifications and exercise, are also strongly advocated globally to alleviate the symptoms of type-2 diabetes. Indeed use of aerobic exercise regimen as an adjuvant therapy results in significant improvement in both insulin resistance and insulin action in major insulin-sensitive tissues [16,18-20]. While a number of molecular/biochemical mechanisms have been put forward to account for the exercise-induced attenuation of hepatic/skeletal muscle insulin resistance and enhanced insulin sensitivity, the underlying mechanism is still not well defined.

Peroxisome proliferator-activated receptor (PPAR)-γ coactivator (PGC)-1α is a highly inducible transcriptional coactivator that controls the transcription of genes involved in a wide variety of biological programs including adaptive thermogenesis, glucose and fatty acid metabolism, oxidative phosphorylation, mitochondrial biogenesis, fiber type switching in skeletal muscle and heart development [21-24]. Under normal and fed conditions, PGC-1α expression is relatively low in liver as compared to tissues such as heart, skeletal muscle, and brown adipose that rely on aerobic metabolism for ATP production [21,22,25]. Its expression in the liver is robustly induced in response to acute food deprivation, insulin resistance or diabetes [25-28], but reduced in obesity [29]. Both loss-of-function and gain-of-function studies have shown that PGC-1α differentially impacts the lipid/glucose metabolism and insulin sensitivity in liver and skeletal muscle. For example PGC-1α overexpression results in hepatic insulin resistance, manifested by higher glucose production and diminished insulin suppression of gluconeogenesis [30]. In contrast, PGC-1α overexpression leads to improved muscle insulin sensitivity, mitochondrial function and insulin regulated glucose metabolism [30-32]. PGC-1α deficiency on the other hand, is accompanied by alterations in energy metabolism in multiple tissues, muscle dysfunstion, hepatic lipid metabolism and insulin signaling and diminished hepatic gluconeogenesis [32-35]. When considered together, these various findings indicate that PGC-1α plays a central role in the regulation of metabolic pathways that control hepatic and skeletal muscle energy metabolism, lipid and glucose homeostasis and insulin action.

Recent studies have shown that exercise increases the expression of PGC-1α in skeletal muscle and it has been suggested that exercise-induced improvements in skeletal muscle oxidative metabolism and insulin sensitivity results, at least in part, through the activation and upregulation of PGC-1α [36-40]. However, it is not clear whether exercise-induced improvements in insulin regulated hepatic lipid and glucose metabolism is also linked to the modulation of PGC-1α expression in the liver. Furthermore, it is not clear whether or not activation and upregulation of PGC-1α occurs uniformly in all skeletal muscle types or restricted to certain fiber types. To address these questions, we have utilized a Wistar rat model of endurance training. The data presented here indicate that exercise selectively upregulates the protein expression of PGC-1α in high-oxidative fast (plantaris) skeletal muscle type. No changes in the PGC-1α protein expression, however, were noted in either the liver or a representative of slow oxidative (soleus) skeletal muscle type. These latter results raised the possibility that exercise-induced improvements that occur in hepatic lipid and glucose metabolism and insulin sensitivity are not fully dependent on the activation and participation of PGC-1α.

## Methods

### Animals

All experimental protocols were approved by the Ethics Committee for Review of Research Projects, of the Hospital of Clinics of the School of Medicine of the University of Sao Paulo. Male Wistar rats, (188.82 ± 2.77 g; 5 wk of age) were obtained from USP (Sao Paulo, Brazil) and kept in an environmentally controlled room (22 ± 1°C) with a 12-h light (0700-1900) and dark (1900-0700) cycle. Animals had free access to food (Rat Laboratory Chow, Nuvilab CR-1) and water. After 1 week of acclimation, rats were assigned to either a sedentary (n = 13) or exercise group (n = 11). Exercising rats were housed individually in cages (42 × 38 × 38 cm) equipped with running wheels, each circumrevolution corresponding to 96 cm, and allowed to run at their own pace for 5 weeks. The number of exercise wheel revolutions was recorded continuously with the aid of a digital counter attached to the wheel to quantify running distance. A group of rats housed individually in standard laboratory cages comprised a sedentary group. Both sedentary and exercising rats were weighed twice weekly at 0900.

### Fasting levels of serum insulin and metabolites

At the end of the training period, exercise rats were not allowed to run for 24 h and food was removed from cages of both sedentary and exercising rats at 0800. Rats were weighed and then anesthetized at 1200 with an injection of 50 mg/kg of pentobarbital sodium (Thiopentax - Cristália Chemicals Products and Pharmaceutics Ltd, Sao Paulo, Brazil). A venous sample was obtained from a cut at the tip of the tail for measurement of glucose, insulin and free fatty acid (FFA) concentration as described below.

### Measurement of in vivo insulin action

Insulin sensitivity was evaluated using the insulin suppression test previously described [41,42]. Procedure started at 1200 after 4 h fast. Briefly, rats were anesthetized by an intraperitoneal injection of 50 mg/kg pentobarbital, and right internal jugular was exposed and cannulated for administration of the infusate. Rats received a continuous infusion of glucose (8 mg/kg) and insulin (2.5 mU/kg) for 180 minutes. With this technique, comparable steady state plasma insulin levels are reached in all animals during the last hour of the study. By measuring the steady state plasma glucose concentration during the third hour, it is possible to get a direct assessment of the ability of a fixed concentration of insulin to stimulate glucose uptake in two groups. Steady state plasma glucose and insulin values were calculated from tail blood samples taken at 10-minute intervals during the last 60 minutes of the infusion and expressed as glucose and insulin areas under the curve (AUC).

### Tissue collection

At the end of 5 weeks of exercise training, all animals were subjected to an insulin suppression test. Subsequently, animals were euthanized for tissue collection. The fast-twitch plantaris [43] and slow-twitch soleus [44] muscle and liver tissues were excised, frozen in liquid nitrogen and stored frozen at -80°C until analyzed.

### Biochemical measurements

Serum glucose, nonesterified free fatty acids (NEFAs), and insulin levels were quantified using kits from LABTEST, Diagnostica S.A., Minas Gerais, Brazil, Linco Research Immunoassay, St. Charles, MO, USA and Boehringer Mannheim, Ingelham, Germany, respectively. For some studies, serum levels of NEFAs were also measured by a kit supplied by Wako Pure Chemical Industries Ltd., Osaka, Japan.

### Immunoblotting

Tissue samples were homogenized in lysis buffer containing 100 mM Tris buffer, pH 7.5, 1% SDS, 10 mM EDTA, 100 mM sodium pyrophosphate, 100 mM NaF, 10 mM sodium vanadate, using a Polytron (Brinkmann Instruments, Westbury, NY, USA). Insoluble material was removed by centrifugation (10,000 *g *for 40 min at 4°C). Protein concentration of tissue lysates was determined by the Bradford dye method (Bio-Rad Laboratories Inc., Hercules, CA, USA). Equal amount of protein (75 μg) was separated on 10% SDS-PAGE gels. Proteins were transferred to nitrocellulose membranes (Millipore), blocked overnight with 5% non-fat dry milk in TBS containing 0.02% Tween-20. The nitrocellulose membranes were washed with TBS containing 0.1% Tween-20 (TBS-T) and incubated for 4 h at 22°C with primary anti-PGC-1α antibody (polyclonal antibody of rabbit against amino acids 1-300 promoted mapped near the N-terminal portion of PGC-1 - Santa Cruz Biotechnology, Santa Cruz, CA). Membranes were washed with TBS-T and incubated with appropriate secondary antibody (horseradish peroxidase-conjugated anti-rabbit IgG) for 1 h at room temperature. Bands were visualized using ECL (Amersahm, Piscataway, NJ, USA). Subsequently, visualized protein bands were scanned, photographed, and analyzed by optical densitometry with Scion Image for Windows (Scion Corporation, National Institutes of Health, Bethesda, MD, USA). Gel-loading of samples was detected by re-probing membrane blots with an anti-β-actin monoclonal antibody (1:1000 dilution; Santa Cruz Biotechnology, Santa Cruz, CA). All data were expressed as an arbitrary unit per unit of total protein mass.

### Statistical analysis

Data are expressed as mean ± SE. Differences between exercise and sedentary groups were determined by an unpaired Student's *t*-test. *P *values < 0.05 were considered significant.

## Results

**Figure 1:**
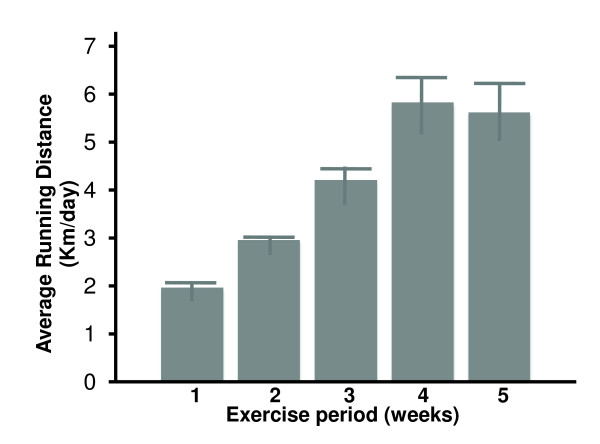
**Average daily voluntary running distance for rats for each week is shown**. Values are presented as mean ± SE, n = 13.

Rats housed in the exercise wheel cages demonstrated almost linear increases in running activity with advancing time reaching to maximum value around 4 weeks (Figure 1). On an average, the rats ran a mean (Mean ± SE) of 4.102 ± 0.747 km/day. Rats weighed 188.82 ± 2.77 g before the start of the exercise regimen. Weight gain over the 5-week period was similar in control and exercise groups with a final weight of 375.68 ± 5.30 g in control (sedentary) as compared with 355.85 ± 9.51 g in the rats allowed to run spontaneously (Table 1). In contrast, exercising rats consumed significantly more food as compared to sedentary controls (*P *< 0.001) in order to meet their increased caloric requirement.

**Table 1:** Mean body weight and food intake

Group	Control	Exercise
Body weight (g)	375.68 ± 5.30	355.85 ± 9.51
Food intake (g/day)	27.11 ± 0.58	36.48 ± 1.03

Results are Mean ± SE

The effects of exercise training on basal plasma glucose, insulin and FFA concentrations are listed in Table 2. Mean plasma insulin concentrations were lower in the exercise-trained rats (*P *< 0.001) as compared to sedentary controls. Likewise, exercise also attenuated the circulating levels of FFA (*P *< 0.006 *vs *control). In contrast, exercise-trained rats were able to maintain glucose levels equal to that of control rats (Table 2).

**Table 2:** Mean basal plasma glucose, free fatty acids (FFA) and insulin concentrations

Group	Control	Exercise
Plasma insulin (ng/dl)	1.45 ± 0.14	0.70 ± 0.12*
Plasma glucose (mg/dl)	122.40 ± 2.62	117.60 ± 3.69
Plasma FFA (mEq/dl)	1.61 ± 0.11	1.12 ± 0.11**

Results are Mean ± SE

*P = 0.001 *vs *control; **P = 0.006 *vs *control

**Figure 2:**
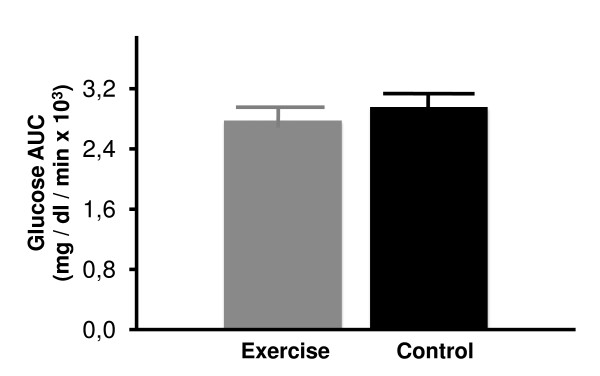
**Steady state plasma glucose (SSPG) concentrations in control sedentary and 5-week exercise-trained rats**. Values are mean ± SE. n = 12-13 rats/group.

**Figure 3:**
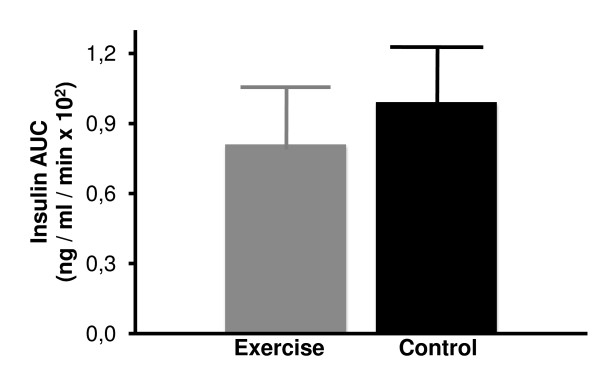
**Steady state plasma insulin (SSPI) concentrations in control sedentary and 5-week exercise-trained rats**. Values are mean ± SE with n = 12-13 rats/group.

Steady state plasma glucose (SSPG) and insulin (SSPI) concentrations of the two groups are shown in Figures 2 and 3. Mean steady state plasma glucose concentrations were not significantly different in sedentary control rats as compared to exercise-trained animals (Figure 2). Similarly, steady state plasma insulin concentrations were also comparable among the two groups (Figure 3). Since SSPG levels were not changed in response to exercise, it is clear that insulin-stimulated glucose uptake in insulin-sensitive normal rats was not further impacted by exercise training.

**Figure 4:**
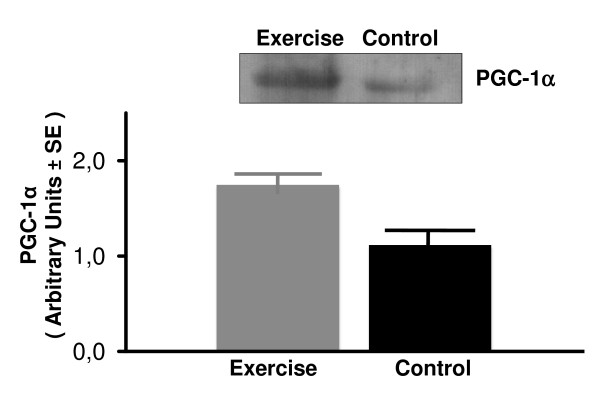
**Total PGC-1α protein content in plantaris muscle assessed by Western blotting in sedentary control and exercised rats**. Values are mean ± SE with n = 8 rats/group. *P < 0.001 vs sedentary control.

**Figure 5:**
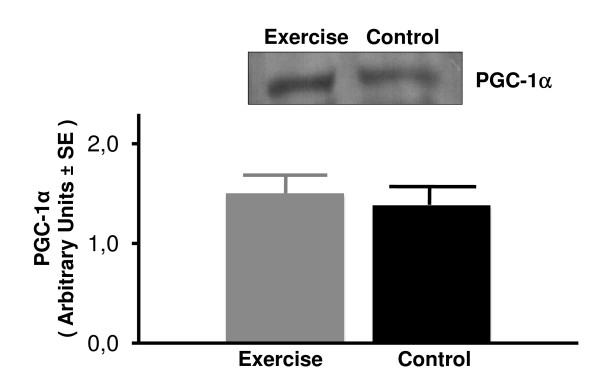
**Total PGC-1α protein content in soleus muscle assessed by Western blotting in sedentary control and exercised rats**. Values are mean ± SE with n = 8 rats/group.

**Figure 6:**
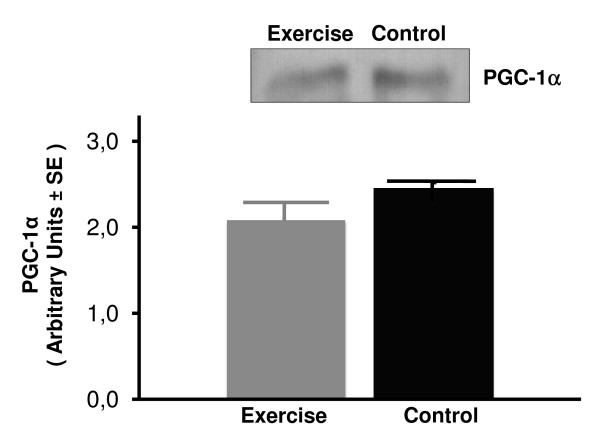
**Total PGC-1α protein content in liver tissue assessed by Western blotting in sedentary control and exercised rats**. Values are mean ± SE with n = 8 rats/group.

The effects of exercise training on the PGC-1α protein expression in soleus and plantaris skeletal muscles and liver as measured by Western blotting are shown in Figures 4, 5, 6. Equal loading of protein was confirmed by probing the membrane blots with an anti-actin monoclonal antibody (data not shown). Plantaris PGC-1α protein expression increased significantly from a 1.11 ± 0.12 in the sedentary rats to 1.74 ± 0.09 in exercising rats (*P *< 0.001) (Figure 4). However, exercise had no effect on PGC-1α protein content in either soleus muscle or liver tissue (Figures 5 and 6). These results indicate that exercise training selectively up regulates the PGC-1α protein expression in the high-oxidative fast skeletal muscle type such as plantaris muscle.

## Discussion

As eluded above, PGC-1α has now emerged as a master regulator of several major metabolic pathways, including adaptive thermogenesis, glucose and fatty acid metabolism, oxidative phosphorylation, mitochondrial biogenesis, fiber type switching in skeletal muscle, and heart development [21-26]. Numerous studies also indicate that PGC-1α is a key factor involved in the regulation of insulin sensitivity [30-32,35,40] and adaptive responses to regular endurance exercise, leading to enhanced oxidative capacity of the skeletal muscle, and consequently, increased capacity for both lipid and carbohydrate utilization [36,38,39,45]. However, less is known about the potential beneficial actions of PGC-1α as a mediator of exercise-induced improvement in hepatic insulin sensitivity and how exercise impacts PGC-1α expression in the liver. Thus, the current study was initiated with an overall goal to assess the relative effects of exercise on the PGC-1α protein expression in two representatives of slow oxidative (soleus) and high-oxidative fast (plantaris) muscle types as well as in the liver and its relevance to whole body insulin sensitivity and circulating insulin and fatty acid levels. The rationale for the present study was based on our previous findings that exercise training enhances insulin sensitivity in rats and that this effect is accompanied by a reduction in circulating insulin concentration [41,46,47]. This phenomenon has been observed in normal rats [41,46,47] as well as rats fed diets enriched with glucose, fructose or sucrose [48]. The results of the current experiments not only confirm our previous observations that exercise training attenuates the plasma levels of insulin, but also lead to a significant reduction in the circulating levels of free fatty acids. More importantly, in the present study, we have demonstrated that exercise training selectively upregulates the PGC-1α expression in high-oxidative fast plantaris muscle, but have no effect on its expression in either slow oxidative soleus muscle or the liver.

It is clear from the data presented that plasma FFA levels were lower in exercise trained chow fed normal rats. However, we cannot tell if lower FFA levels noted in exercise trained rats were due to increased fatty acid oxidation by liver and/or skeletal muscle or simply the negative impact of running on the hormone-sensitive lipase (HSL) and adipose triacylglycerol lipase (ATGL), the two key enzymes involved in lipid hydrolysis and release of FFA from adipose tissue depots. The latter possibility is unlikely given that optimal functioning of both HSL and ATGL is necessary in maintaining adequate supply of FFA to sustain whole body substrate metabolism in response to exercise [49,50]. A more likely possibility that needs to be considered is that exercise training may lower protein expression of liver MTP, a molecule that is essential for VLDL assembly and secretion [51], and in turn causes increased channeling of FFA for their disposal through oxidation. Indeed, it has been shown that exercise training reduces VLDL synthesis and/or secretion at least in high-fat fed rats most likely through modulation of MTP [52]. Whether or not exercise-induced negative regulation of MTP-VLDL ultimately turns out to be responsible for the attenuation of dyslipidemia will depend on the results of future studies, but the observed reduction in circulating FFA levels supports the view that this possibility is worthy of continued consideration.

Exercise is known to cause an acute and transient increase in skeletal muscle PGC-1α gene expression [36,37,53,54]. Conversely, cessation of physical activity reduces the skeletal muscle PGC-1α mRNA expression [54]. It is suggested that exercise-increased levels of skeletal muscle PGC-1α leads to enhanced mitochondrial electron transport activities that enables cells to meet rising energy demand. We show here that spontaneous wheel running up regulates PGC-1α protein expression only in plantaris muscle, but not in soleus muscle. These results are in agreement with a previous study showing that voluntary wheel running of female ICR mice was associated with a selective increase in PGC-1α protein expression in plantaris muscle [55]. However, we cannot tell if the enhanced PGC-1α protein expression in plantaris muscle noted in the running rats was due to the effect of exercise training or simply the reflection of plantaris muscle hypertrophy [56]. Moreover, it is also unclear whether steady state increases in PGC-1α expression results from increased de novo synthesis, decreased degradation or both. Clearly, further studies will be necessary to investigate these possibilities.

The observation that exercise selectively up regulates the expression of PGC-1α only in the plantaris muscle points to a potential dissociation between exercise-mediated modulation of PGC-1α and improvements in skeletal muscle oxidative metabolism. Most of the previous studies have shown a positive correlation between exercise-induced changes in total skeletal PGC-1α mRNA and mitochondrial electron transport activities [36,37,53,54,57,58]. Our results raise the possibility that contrary to the previous findings, PGC-1α may not function as a global regulator of oxidative metabolism in response to exercise. Instead, we suggest that exercise-mediated improvement in skeletal muscle oxidative metabolism may involve improved PGC-1α action in specific muscle types (e.g., oxidative muscle such as plantaris muscle) or exercise-induced recruitment other biologically active compounds leading to increased functional efficacy of PGC-1α [59-65]. In addition, exercise-induced potential alterations in acetylation, phosphorylation, and/or methylation status of PGC-1α could also positively impact its function (65). Furthermore, since we have used 5 weeks of voluntary wheel running exercise regimen, there remains the possibility that we may have missed any potential transient increases in PGC-1α in soleus muscle studied here. Indeed, exercise is known to cause an acute and transient increase in PGC-1α gene expression in various skeletal muscle types [36,37,53,54,66] The follow-up studies are underway to assess the impact of short- and long-term exercise training on the expression of PGC-1α not only in these two muscle types, but also in a variety of other skeletal muscle types with differing fiber composition [67]. Also, experiments are in progress to determine effects of short-term and long-term exercise treatment regimen on the acetylation, phosphorylation, and/or methylation status of PGC-1α in specific skeletal muscle types as well as in liver.

Previous studies have shown that expression in the liver is robustly induced in response to acute food deprivation, insulin resistance or diabetes [25-28], but reduced in obesity [29]. Both loss-of-function and gain-of-function studies have shown that PGC-1α over expression results in hepatic insulin resistance, while PGC-1α deficiency is accompanied by alterations in hepatic energy and lipid metabolism, lipid accumulation (steatosis), insulin signaling and diminished hepatic gluconeogenesis [33-35]. These observations have led to a general thinking that hepatic PGC-1α may function as a negative modulator of insulin sensitivity, and lipid and glucose metabolism. Despite our expectations that exercise-mediated improvement in hepatic oxidative metabolism [68-70] should accompany a significant decline in PGC-1α expression, we found no change in its expression at the end of 5-weeks of exercise training of rats. It remains possible that at some point beyond 5-weeks of training, PGC-1α expression may be significantly altered or that exercise may indirectly influence PGC-1α through modulation of SIRT1 which serves as a regulator of PPARα-PGC-1α directed gene expression in liver [65]. Likewise, it is possible that we may have missed a transient change (decrease) in PGC-1α expression during the early phase of the exercise treatment regimen. Moreover, we tested the effect of exercise only in male Wistar rats, in which animals were subjected to voluntary wheel running starting at 5 weeks of age. Thus, our findings do not preclude the possibility that other important factors such as strain, sex, and age at the start of the exercise regimen may render animals more responsive to exercise-induced changes in PGC-1α expression in the liver.

In conclusion, results of the present study indicate that 5 weeks of voluntary wheel training exercise results in a significant reduction in circulating levels of insulin and FFA. Western blot analysis indicated that exercise selectively up regulated the PGC-1α protein expression, a major mediator of exercise-induced improvements in skeletal muscle oxidative metabolism and insulin sensitivity, in the high-oxidative plantaris muscle. In contrast, exercise training had no effect on the PGC-1a expression in either the slow-oxidative soleus muscle or liver. Based on these findings, we conclude that PGC-1α plays a minor role in exercise-mediated improvements in insulin resistance (sensitivity) and lowering of circulating FFA levels.

## List of abbreviations used

SSPG: steady state plasma glucose; SSPI: steady state plasma insulin; AUC: area under the curve; FFA: free fatty acid; PGC-1α: peroxisome proliferator-activated receptor (PPAR)-γ coactivator.

## Competing interests

The authors declare that they have no competing interests.

## Authors' contributions

RM, RF, MS and DR carried out various aspects of the experiments summarized in this manuscript. RS conceived of the study and BW together with RS participated in the design and coordinated the performance of the experiments. SA provided technical advice on this project and RS and SA drafted the manuscript. All the authors read and approved the final manuscript.
